# Mobile educational follow-up application for patients with peripheral arterial disease*

**DOI:** 10.1590/1518-8345.2693-3122

**Published:** 2019-01-14

**Authors:** Cristiane Baldessar Mendez, Nádia Chiodelli Salum, Cintia Junkes, Lucia Nazareth Amante, Carlos Mauricio Lopes Mendez

**Affiliations:** 1 Universidade Federal de Santa Catarina, Hospital Universitário Polydoro Ernani de São Thiago, Florianópolis, SC, Brazil.; 2 Universidade Federal de Santa Catarina, Centro de Ciências da Saúde, Florianópolis, SC, Brazil.; 3 Mendez & Baldessar, Curitiba, PR, Brazil.

**Keywords:** Peripheral Arterial Disease, Mobile Applications, Nursing Care, Educational Technology, Information Technology, Continuity of Patient Care, Doença Arterial Periférica, Aplicativos Móveis, Cuidados de Enfermagem, Tecnologia Educacional, Tecnologia da Informação, Continuidade da Assistência ao Paciente, Enfermedad Arterial Periférica, Aplicaciones Móviles, Atención de Enfermería, Tecnología Educacional, Tecnología de la Información, Continuidad de la Atención al Paciente

## Abstract

**Objective:**

to describe the development of a prototype mobile educational application for nursing follow-up aimed at patients diagnosed with peripheral arterial disease.

**Method:**

a prototype-based technological production study. The construction followed the contextualized instructional design model using two steps: analysis and design and development.

**Results:**

the pedagogical content of the application was based on a survey of needs of patients with Peripheral Arterial Disease and treatments recommended in the literature. The prototype developed contained concepts, risk factors, signs and symptoms, treatment, importance of medications and their side effects, frequent doubts, necessary health care, and follow-up of patients by monitoring the evolution of the cicatricial process of lesions and possible complications, clarification of doubts and stimulus for continuation of treatment.

**Conclusion:**

the use of health applications is a technological tool with the potential to improve the follow-up of patients regarding the progress of the disease and self-care, monitoring of risk factors, co-participation of the patient in the treatment, family participation, as well as planning of individualized care, and cost reduction for the health system.

## RESUMO

**Objetivo:**

descrever o desenvolvimento de um protótipo de aplicativo móvel educativo e de *follow up* de enfermagem para pacientes com diagnóstico de doença arterial periférica.

**Método:**

estudo de produção tecnológica do tipo prototipagem, a construção seguiu o modelo do *design* instrucional contextualizado, utilizando duas etapas: análise e *design* e desenvolvimento.

**Resultados:**

o conteúdo pedagógico do aplicativo baseou-se no levantamento das necessidades dos pacientes com Doença Arterial Periférica e nos tratamentos recomendados pela literatura. O protótipo desenvolvido foi composto por conceitos, fatores de risco, sinais e sintomas, tratamento, importância dos medicamentos e seus efeitos colaterais, dúvidas frequentes, cuidados necessários com a saúde e o *follow up* dos pacientes por meio da monitorização da evolução do processo cicatricial das lesões e possíveis complicações, esclarecer dúvidas e estimular a continuidade do tratamento.

**Conclusão:**

o uso do aplicativos em saúde é uma ferramenta tecnológica com potencial para melhorar o acompanhamento de pacientes quanto à evolução da doença e autocuidado, no acompanhamento dos fatores de risco, coparticipação no seu tratamento, a participação familiar, bem como planejar um cuidado individualizado e redução de custos para o sistema de saúde.

## RESUMEN

**Objetivo:**

describir el desarrollo de un prototipo de aplicativo móvil educativo y de *follow up* de enfermería para pacientes con diagnóstico de enfermedad arterial periférica.

**Método:**

estudio de producción tecnológica del tipo prototipación, la construcción siguió el modelo del design instruccional contextualizado, utilizando dos etapas: análisis y *design* y desarrollo.

**Resultados:**

el contenido pedagógico del aplicativo se basó en el levantamiento de las necesidades de los pacientes con Enfermedad Arterial Periférica y en los tratamientos recomendados por la literatura. El prototipo desarrollado fue compuesto por conceptos, factores de riesgo, señales y síntomas, tratamiento, importancia de los medicamentos y sus efectos colaterales, dudas frecuentes, cuidados necesarios con la salud y el *follow up* de los pacientes por medio del monitoreo de la evolución del proceso cicatricial de las lesiones y posibles complicaciones, aclarar dudas y estimular la continuidad del tratamiento.

**Conclusión:**

el uso de aplicativos en salud es una herramienta tecnológica con potencial para mejorar el acompañamiento de pacientes como a la evolución de la enfermedad y autocuidado, en el acompañamiento de los factores de riesgo, coparticipación en su tratamiento, la participación familiar, así como planificar un cuidado individualizado y reducción de costos para el sistema de salud.

## Introduction 

Mobile web or mobile connection entered the market in the year 2000 and is defined as wireless technology to access information and applications anywhere and anytime through mobile devices such as cellphones, smartphones and tablets^1^.

Mobile computing can be used in many health care areas such as support to medical and nursing diagnosis, decision making, electronic medical record of the history of medical examinations, diagnoses and consultations, assessment of nursing workload, drug stock control, bed management, besides the focus on patient support with consultation/return reminders via Short Message Service (SMS), remote monitoring, pain management, post-discharge follow-up, reduction of ambulatory consultations in long-term treatments, stimulation to adherence to treatments and healthy life^2^ ^-^ ^3^.

In this context, there are studies that highlight the use of mobile applications via smartphones for postoperative follow-up in order to identify possible surgical complications. These follow-up takes place through photos, guidance and clarification of doubts regarding the use of medications, monitoring of potential adverse events such as postoperative pain and complications^3^ ^-^ ^4^.

Monitoring patients with chronic diseases is especially important to reduce costs with surgical interventions and complications that can be avoided. One example is Peripheral Arterial Disease (PAD), a chronic obstructive process mainly caused by atherosclerosis. 

The regions of the body most affected by PAD are the lower limbs. However vascular disease is responsible for cardiovascular and cerebrovascular complications. The reduced blood supply in peripheral arteries is responsible for symptoms of intermittent claudication (IC) and resting ischemic pain^5^ ^-^ ^6^.

In Brazil, the annual estimate of PAD is 0.053% of the population of men aged 55-74 years and women aged 65-74 years^7^. The asymptomatic evolution of PAD can reach up to 70 to 80% of patients. This delays and hinders early diagnosis. Early diagnosis, in turn, is essential for the identification of risk factors and changes in lifestyle in order to improve the effectiveness of treatment, reduce the risk of complications such as ulcers and early amputations, and ensure the quality of life of patients^8^.

Many PAD patients are often observed to make telephone calls to the inpatient unit to resolve their doubts, to confirm that the guidelines received are correct, or they go to the inpatient unit to seek help.

Communication and information via Web 3.0 and Mobile Applications (Apps) in the health area can be a facilitator of the health services’ functioning dynamics. They can reduce or avoid displacement, reduce demands on the health network, minimize aggravation due to lack of therapeutic support, and facilitate referral and counter-referral, thus improving the link with the Health System and with a care planning focused on the patient’s need.

Web 3.0 consists in the generation that allows the organization, reuse and replication of data about the user at any time and anywhere, being able to interact through the analysis of information provided by the user^9^.

Health education aims to increase people’s autonomy and ability to intervene in their own lives. Information and Communication Technologies (ICTs), in turn, have the potential to contribute significantly to improving access to quality services and at the same time, reduce costs^10^. Thus, the strategy of follow-up through mobile applications can contribute to the qualification of health care for patients undergoing PAD treatment.

In this perspective, this study had as guiding question: What contents should be included in an educational mobile application to follow-up PAD patients? The objective of this study was to describe the development of a mobile educational follow-up nursing application for people diagnosed with peripheral arterial disease designed with the aim to modify behavioral risk factors such as inadequate diet, sedentary lifestyle, smoking, overweight and non-compliance with the prescribed drug therapy. Follow-up can be done through the exchange of messages, clarifying doubts that may interfere in the control of the disease.

## Method

This is a prototypying technology production aimed at building an educational mobile application by scientific rigor. The nursing treatment provided in a teaching hospital in southern Brazil in a surgical clinic unit that serves patients with peripheral arterial disease was used as reference for this study, from May to November 2017. 

The development of the study was organized and guided according to the Contextualized Instructional Design (CID) model^11^, which consits in providing tools and resources to reach learning needs. This model consists of 4 stages: analysis: involving the survey of the learning needs, the definition of the instructional objectives that are to be achieved, and the research of the limitations involved; b) design and development: involving the planning of the instruction and elaboration of instruments and tools to be used; c) implementation: including training on the use of educational technology tools and resources and the realization of the teaching event or learning situation per se; and, finally, d) evaluation: including the evaluation of specialists as to the contents, didactic resources, interface of the environment, and maintenance^11^ ^).^

In turn, the application was developed in two stages: 1) analysis and 2) design and development. They are described below:

Stage I - analysis: in this stage, the needs of patients with PAD were identified^12^, particularly those related to the difficulties and doubts that may arise after hospital discharge. An integrative literature review was conducted to identify the evidence of currently proposed treatments to prevent the disease progression and promote better quality of life. And, lastly, the technological prospection of similar ideas, carried out through the analysis of similar applications (Apps) already existing in the virtual stores. The analysis sought to describe the current situation in intellectual production focused on health education and home-based follow-up.

The review and synthesis of knowledge was carried out in the PUBMED, MEDLINE, CINAHL, LILACS and SCIELO databases, using the following Descriptors in Health Sciences (DeCS): peripheral arterial disease, therapeutics, complications and nursing care, complemented by keywords related to each descriptor: peripheral obstructive arterial disease, arterial disease, peripheral arteriopathies, peripheral occlusive arterial disease, therapy, treatment, complications and adherence to treatment. The inclusion criteria were: scientific articles, national and international guidelines on treatment for patients diagnosed with PAD, published between 2014 and 2017. The exclusion criteria were: articles discussing medical techniques, case studies, experience reports, and integrative reviews.

Stage II - design and development: this stage is represented by the elaboration of the contextualized instructional content and by the methodology of development of the educational App to *follow-up* patients with diagnosis of PAD. This stage was organized in two moments, described as follows:

First Moment - *Design*: This was the moment of definition of the educational content, of the screen navigation structure, as well as the visual and functional organization and its typography, that is, the composition of the *layout* along with perception issues such as font type, font size, spacing, colors and positioning of images, figures and animations. For this activity, a volunteer information technology professional contributed through consulting/support at this moment.

This stage was based on the data obtained in the stage I. The pedagogical content was composed of concepts of the disease, information on risk factors, diagnosis, treatment and guidance for prevention of complications.

The necessary language to effectively convey the information offered in the App was also defined. The Sketch tool (The digital design toolkit) was used for building the mockups. This tool allows the diagramming of the screens and of their navigation flow to ANDROID and IOS platforms.

Mockups are basically the screen layouts that serve to directly show the final architecture and navigation flow of the application, according to the specifications.

Second Moment - The development phase corresponded to the production of the learning object itself, that is, the coding of the application in computational language and storage in the chosen platform. This step was conducted by a senior volunteer programmer who used hybrid technology that serves multiple platforms. The application interface was developed using the Javascript and HTML5 programming languages (HyperText Markup Language), IONIC v3 and Angular v4 frameworks, and the SQLite and Firebase databases for storage of information, making the development more dynamic and more compatible with the ANDROID and IOS platforms. The Cascading Style Sheets (CSS) tool was also used to configure and organize the screens, images, buttons, fonts, and colors.

The study was submitted to the Human Research Ethics Committee of the Federal University of Santa Catarina under the number 76825717.8.0000.0121, and authorization was requested from the author^12^ to use the unpublished data of her study.

## Results

The results are presented according to the construction steps:

**Figure 1:**
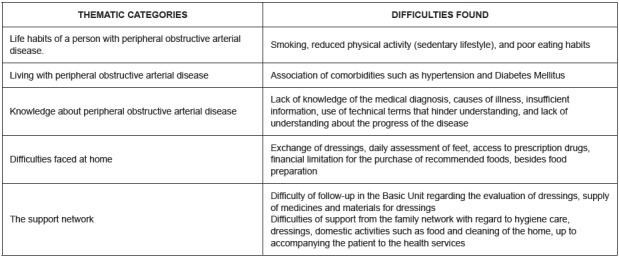
Difficulties faced by patients with Peripheral Arterial Disease by Martins^12^. Florianópolis, SC, Brazil, 2017

Stage I: doubts of PAD patients were identified based on a previous study^12^. The doubts were related to knowledge about the disease, life habits, difficulties at home after hospital discharge, and the necessary guidelines for the continuation of the proposed treatment to return to the community (Figure 1).

The integrative review listed studies on good practice in the treatment of PAD. Seventy-nine retrieved articles were within the scope of the review, of which 23 were published in 2014, 25 in 2015, 21 in 2016 and 10 in 2017.

As for the professional category, 88.6% (70) were publications of medicine, 3.7% (3) of nursing, 3.7% (3) of physiotherapy and 2.5% (2) of psychology. Regarding the theme approached, 37.9% (30) treated the endovascular treatment, 8.8% (7) open vascular surgery, 21.5% (21) pharmacological treatment, 8.8% (7) control of risk factors, 12.6% (10) supervised physical exercise and 1.2% (1) treatment of lower limb injury.

Studies addressing the evaluation of lower limb vascular surgery and endoprostheses examined the impact of procedures on intermittent claudication, reduction of PAD complications such as amputations, durability and efficacy, decrease in Ruthenfort index, healing rate of wounds, quality of life/morbidity and mortality, and financial impact between procedures^13^ ^-^ ^21^.

Studies addressing drugs evaluated the use of histamines, angiotensin converting enzyme (ACE), antiplatelet, angiotensin converting enzyme inhibitors (iACE), or angiotensin receptor blockers (ARB) in the treatment of PAD, in the prevention of cardiovascular adverse events and lower limb complications^22^ ^-^ ^27^.

Other studies present the contribution of physical exercise as a therapy to improve walking distance without presenting intermittent claudication and an increase in the Ankle-Brachial Index (ABI) in the short- and medium-term associated with pharmacological treatment and/or intervention^28^ ^-^ ^31^. 

As for therapies recommended for the treatment of PAD, we evaluated the association of eight therapies: antiplatelet agents, statins, angiotensin converting enzyme inhibitors, blood pressure control, lipid control, diabetic glycemic control, cessation of smoking, and body mass index. It was also evidenced that the involvement of vascular surgeons, family physicians, and patients with PAD favored a positive result for reduction of cardiovascular risk and lower limb complications^25^.

**Figure 2:**
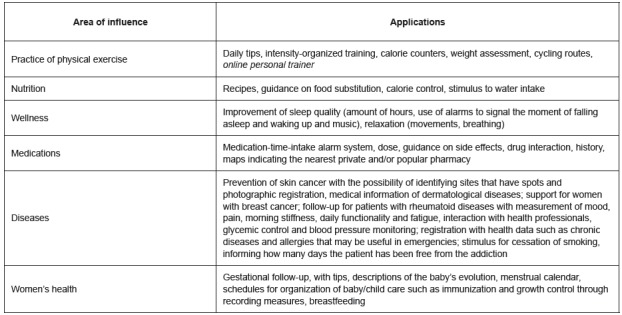
Technological prospecting data, 2017

At this stage, we also sought to carry out technological prospecting by analyzing similar Apps aimed at the pursuit of a healthy life for patients and/or individuals. We evaluated 30 Apps aimed at several segments (Figure 2).

No App was found targeting patients with PAD or arterial diseases. It is noteworthy that most Apps are available in English, and have no version translated into Portuguese.

Stage II: design and development. The instructional content of the application was developed to follow-up based on the surveyed needs of patients with PAD and the treatments recommended by the identified literature. Based on this analysis, the content to be made available in the application was defined. The pedagogical content was composed of concepts, risk factors, signs and symptoms, treatment, importance of medications and their side effects, frequently asked doubts, and necessary health care measures. Also included in the App was the folow-up of patients and monitoring of the evolution of the cicatricial process of the lesions with referral of photos of the lesion, through messages sent directly from patient to professional, with the possibility of filling a form describing the characteristics of the lesion (smell, tissue, exudation) with the purpose of identifying possible complications, clarifying doubts and stimulating the continuation of treatment.

In this process, the professional can follow-up the progress of the patient and provide health education by sending guidelines on the doubts that have not been resolved with the content presented in the application.

Besides the focus on the disease, there were also concerns regarding lifestyle changes with a link to physical exercise monitoring, by counting of steps (pedometer), in order to signal the course walked in daily basis and the activity status. There is also a guide to register medicines. This guide allows the inclusion of medicines for daily use, dose, medication-time-intake alarm, and a monthly report of the use of medicines indicating possible forgetfulness.

**Figure 3:**
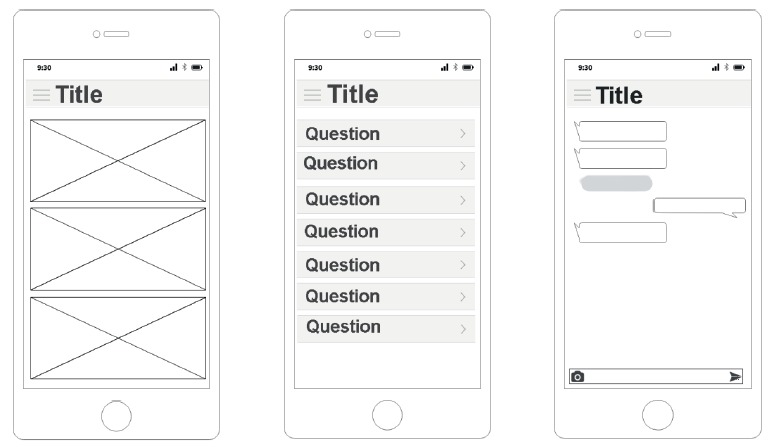
Mockup models. Florianópolis, SC, Brazil, 2017

Mockups were created with the developer, after the composition of the elaborated content. Firstly, a paper draft was made to visualize the possible screens and navigation flows. Then, the mockups were developed in the Sketch tool, which has a comprehensive and integrated library for Wireframe, Material Design, IOS and Web and made it possible to upload the project, that is, bring a detailed idea of the application before starting the current coding (Figure 3).

During the creation of the mockups, the necessary illustrations and some functionalities that were still missing were identified. At least three versions of multi-screen mockups were developed. In each version, new detailed refinements continued to be presented. In the final version, it was already possible to clearly visualize how the application would appear in its layout, as well as in its navigation flow. During the preparation of mockups, the color palette to be used and the text layout were also defined.

The application used a framework development environment which allows the construction of mobile applications using the HTML 5, CSS (Cascading Style Sheets) and JavaScript and Typescript programming languages. Based on a hybrid development, the IONIC provided the coding of a single application, with possibility of use in ANDROID and IOS platforms. The interaction features with the mobile device can be verified in the implementation of the reminder of the medication time and dose, as well as the use of the camera to send images to the digital nurse. Plugins simplify and execute specific functions of the application with greater ease and practicality, and are able to report data about its user such as information produced by the pedometer.

The prototype of the peripheral arterial disease application was developed for the ANDROID and IOS platforms. For its installation, it is necessary to setup to allow the installation from stores (play store and Apple store) or through availability by the health service.

**Figure 4:**
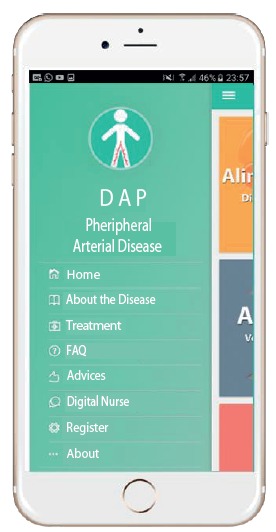
Menu screen

Figure 4 shows the initial screen of the application with the active menu. This menu has eight icons that discuss the disease, the treatment, frequently asked questions, and the role of the application.

During the textual reading, the patient can visualize links with illustrations representing the proposed subject, the step by step of how to perform the daily evaluation of the feet and how to correctly perform the dressing, preventing complications and contamination of the surgical wound.

The icon to exchange messages allows the patient to send his questions and request an evaluation of the evolution of the lesion through the picture sent to the specialized professional that is linked to the application/institution.

On the initial page, there are still three managers: physical activity (pedometer with activity level classification), which reminds the patient of his level of daily activity. The medication control, according to previous registration of medicines to be taken, allows including the picture of the medication to facilitate the identification of each drug and its dosage. The patient receives notifications in the mobile device according to the predetermined schedules and has the option to register whether or not he took the medication, generating a monthly score of his adherence to the treatment. The healthy diet aims to enable search links of easy low-cost recipes that contribute to a balanced diet.

## Discussion

Key points to ensure a tool that provides health education and effective and safe follow-up for patients with peripheral vascular disease were listed for the creation of the App. The instructional content was based on the identification of needs/difficulties faced in the home of the patients, according to the recommended therapies, as well as on a platform that is consistent with an execution with comprehensive testing resources, intuitive usability, data transfer protocols, secure storage, and network identification capacity. The application successfully meets the demand of patients, because it founded on their needs. 

Studies show that individual care planning and health education carried out by professionals lead to improvements in physical and psychological health indicators and increased ability of self-management of the patients’ health conditions^32^. In this sense, the follow-up and the idea of ​​knowing the patients better, being closer, aims to trigger the effectiveness of self-care practices.

In the survey of the needs it was possible to identify as difficulties for the continuation of treatment the care measures with the wound, especially the dressing, the control of risk factors such as Diabetes Mellitus, Systemic Hypertension, dyslipidemias, weight control, daily evaluation of feet, physical exercise, cessation of smoking, and lack of medication and dressing materials in the Basic Unit.

Abstinence from smoking is seen as a fundamental pillar in the treatment of PAD, since smoking influences the development of cardiovascular complications in three different levels: fat plaque progression, inhibition of endothelial repair processes and inhibition of angiogenesis^33^.

Low purchasing power was listed as a justification for not maintaining a healthy diet, interrupting medication use, and difficulties in hiring a caregiver^12^.

A study^34^ shows the difficulties patients face to adhere to a healthy diet, including their preference for carbohydrates and sugars instead of fruits and vegetables, the ease of acquiring processed foods, which are of low cost, the lack of time to prepare foods, not to mention the wrong idea that healthy eating is not tasty and diet is a sacrifice.

Other studies^35^ ^-^ ^36^ state that socioeconomic characteristics and educational level are not directly associated with adherence to treatment, and highlight the performance of the professional team as a key factor for the understanding about the disease and the search of the patient for the health unit.

Although new technologies have emerged in recent years for the treatment of PAD, there is still a gap related to health education for the prevention and control of risk factors. Brazilian studies evaluating the follow up of patients diagnosed with PAD and the impact of the control of these risk factors on morbidity and mortality and on the quality and life of these patients were not found. However, an application of nursing folow-up to patients submitted to antineoplastic chemotherapy proved to be effective with 100% of approval by patients. The follow-up was carried out by telephone for 28 days and provided clarification of doubts, benefiting the recovery of patients at their homes, as well as increasing safety for family members and caregivers^37^. 

In another study with 136 patients diagnosed with asthma and allergic rhinitis using a mobile platform as a tool for rapid patient-professional interaction through message exchange, health status recording and drug use monitoring demonstrated a considerable impact on outcomes of health and quality of life; there was a decrease in the number of hospital admissions, frequency of medical consultations, and loss of productivity^38^ ^).^

In this sense, nurses are clearly important in the health education of patients, to improve adherence to treatment. Thus, mobile technologies for follow-up are currently useful, easy-to-access, wide-ranging, real-time and low-cost tools.

Advances in the development of mobile technology and health communication have been considered an effective strategy to encourage patients to adopt healthy lifestyles, to provide counseling on the disease, to stimulate self-care, to strengthen conditions for chronic diseases, and to reduce time and cost for patients and for the health system^39^.

A study on the use of mobile applications to stimulate behavior changes such as healthy eating, with increased consumption of fruits and vegetables, and realization of physical exercise, showed a positive association with life habits. However, the study pointed out that the main users of the applications are young people of high socioeconomic power and educational level^40^. In line with these findings, a study with an App for young adults with idiopathic juvenile arthritis encouraged self-management and increased the involvement with health care, besides high levels of acceptability and usability^41^.

A study about the efficacy of cellphone and tablet applications in self-management of symptoms of chronic diseases made it possible to show an improvement in the health status of patients living with symptoms of diabetes mellitus, cardiovascular diseases and pulmonary diseases^42^.

Discussing the application of mobile services, a study^43^ stated that there is a prevalence of mobile health services directed to facilitate the care for the professionals, and just a minority focused on health education and follow-up. The study also stresses the need for further investigation on the effects and cost-effectiveness of mobile technologies to increase the speed of communication between healthcare professionals and patients, combining text messaging and photographs as tools to improve clinical diagnosis.

In the perspective of effectiveness of a user-based software, one of the most important factors is the interface, i.e. the communication between the user and the system; this should be easy to learn and intuitive, and the users should follow the steps with ease^44^ ^).^

The application interface for follow-up of patients with PAD was designed to be simple, comprehensible and with few elements, in order to be pleasant to the eyes and easy to handle for the elderly, the group with the highest prevalence of the disease. The App was built for the IOS and ANDROID platforms, for greater access to patients with the aim of guiding and advising patients in their self-care, as well as providing asynchronous information about wounds and procedures to be taken, reducing costs with displacement and hospitalization.

The main determinants of the control of chronic diseases or even of the quality of life of patients are linked to the control of risk factors ^(^ ^45^. For this reason, we sought to emphasize knowledge about the disease but with a focus on educational, motivational and increased awareness through images, medication reminders, exercise and healthy eating, and text messages to ensure follow-up and provide tranquility as a result of the regular monitoring. This first version of the App is divided into three purposes, namely, health education, motivation/and awareness, and follow-up.

The App will be made available in virtual stores (play store and Apple store) for free as educational content for people interested in understanding PAD better and looking for lifestyle changes. In this case, the App will not have the function of follow-up but rather of education. The tool can only be used for follow-up in accredited institutions that provide qualified professionals to monitor patients linked to the health service. Patients should be guided about the use of the App, the registration for usability, and its functionality as soon as during hospitalization. This close guidance favors the bond between health professional and patient and his/her family during the moments of informing about the disease, the therapy and its effectiveness, in an individualized way, as it is fundamental for adherence to treatment. 

## Conclusion

The use of mobile applications in the health area has the potential to improve outcomes among patients with chronic diseases through improved control of risk factors, stimulation of patient participation in treatment, stimulation of family participation, and stimulation of health care. This is a possibility, that is, the use of applications per line of care, as advocated by the Brazilian health system.

Key points to ensure a tool that provides health education and effective and safe follow-up for patients with peripheral vascular disease were listed for the creation of the App. Many of the difficulties encountered by patients are related to socioeconomic power. This means that prevention is an important way to reduce the cost of medication, hospitalization and displacement. The use of Apps acts as an effective strategy to invest in the quality of life of chronic patients by systematic and rapid follow-up.

The lack of validation and evaluation of the application regarding the presentation of content, functionality, and usability by specialists and users appears as a limitation of the study. It is necessary to measure the impact of the follow-up application for peripheral arterial disease on the health of Brazilians, and also compare the costs of the use of mobile technology in traditional follow-up/consultations. The measurement of the impact of this technology on the lives of these patients and on the sustainability of health services is also important.
